# Supplementary material for: Atomistic Mechanism of Calcium-Mediated Inward Rectification of the MthK Potassium Channel by Solid-State NMR and MD Simulations
Source: J Am Chem Soc. 2025 Dec 1;147(49):45536–48. doi: 10.1021/jacs.5c16155 (PMC12703655; doi:10.1021/jacs.5c16155)
Supplement: Supplementary file 1 [file ja5c16155_si_001.pdf]

Supplementary Information for:

Atomistic Mechanism of Calcium-Mediated Inward Rectification of the MthK Potassium Channel by Solid-State NMR and MD Simulations

Authors:

Carl Öster(a)[#], Reinier de Vries(b)[#], Juan Li(a,c), Denis Qoraj(a), Sascha Lange(a), Chaowei Shi(a,d), Wojciech Kopec(b,e)*, Bert L de Groot(b)*, Adam Lange(a,f)*

Affiliations:

(a) Research Unit Molecular Biophysics, Leibniz Forschungsinstitut für Molekulare Pharmakologie (FMP), Robert-Rössle-Straße 10, 13125 Berlin, Germany

(b) Computational Biomolecular Dynamics Group, Max Planck Institute for Multidisciplinary Sciences, Am Fassberg 11, 37077 Göttingen, Germany

(c) MOE Key Lab for Cellular Dynamics, School of Life Sciences, Division of Life Sciences and Medicine, University of Science and Technology of China, Hefei 230026, China

(d) Hefei National Research Center for Interdisciplinary Sciences at the Microscale, University of Science and Technology of China, Hefei, Anhui 230026, China

(e) Department of Chemistry, Queen Mary University of London, 327 Mile End Road, London E1 4NS, United Kingdom

(f) Institute of Biology, Humboldt-Universität zu Berlin, Invalidenstraße 42, 10115 Berlin, Germany.

[#]These authors contributed equally to this work.

*Correspondence should be addressed to W.K. (w.kopec@qmul.ac.uk), B.L.d.G. (bgroot@mpinat.mpg.de), or A.L. (alange@fmp-berlin.de)

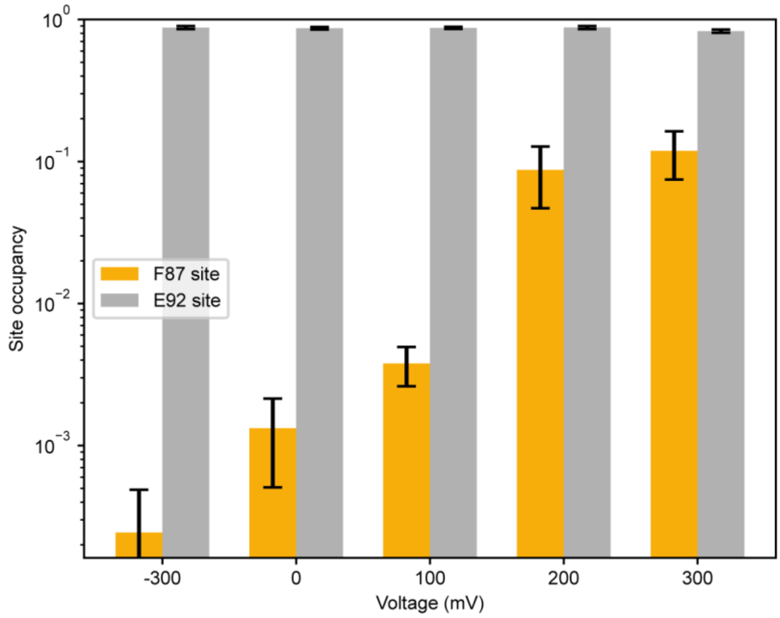

Figure S1. Voltage dependent occupancy of Ca^{2+} binding sites. The F87 site is defined as between 0 and 0.8 nm from the T59 Cα, the E92 site as between 1 and 2 nm from the T59 Cα. Error bars represent standard error of the mean over 10 independent replicas.

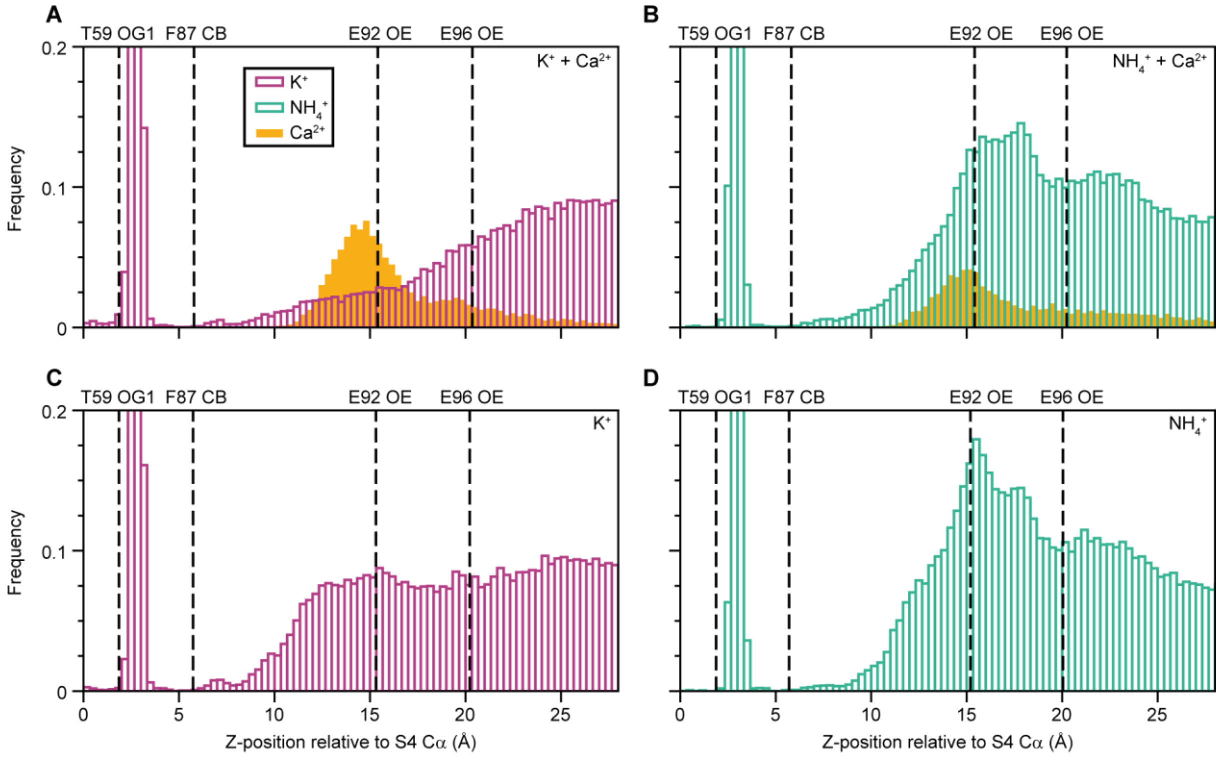

Figure S2. Ca^{2+} binding in the MthK cavity under negative voltage. Ion densities in the cavity relative to T59 from MD simulations under -300 mV over 10 replicates (A) KCl + $CaCl_2$,(B) NH_4Cl + $CaCl_2$,(C) KCl,(D) NH_4Cl.

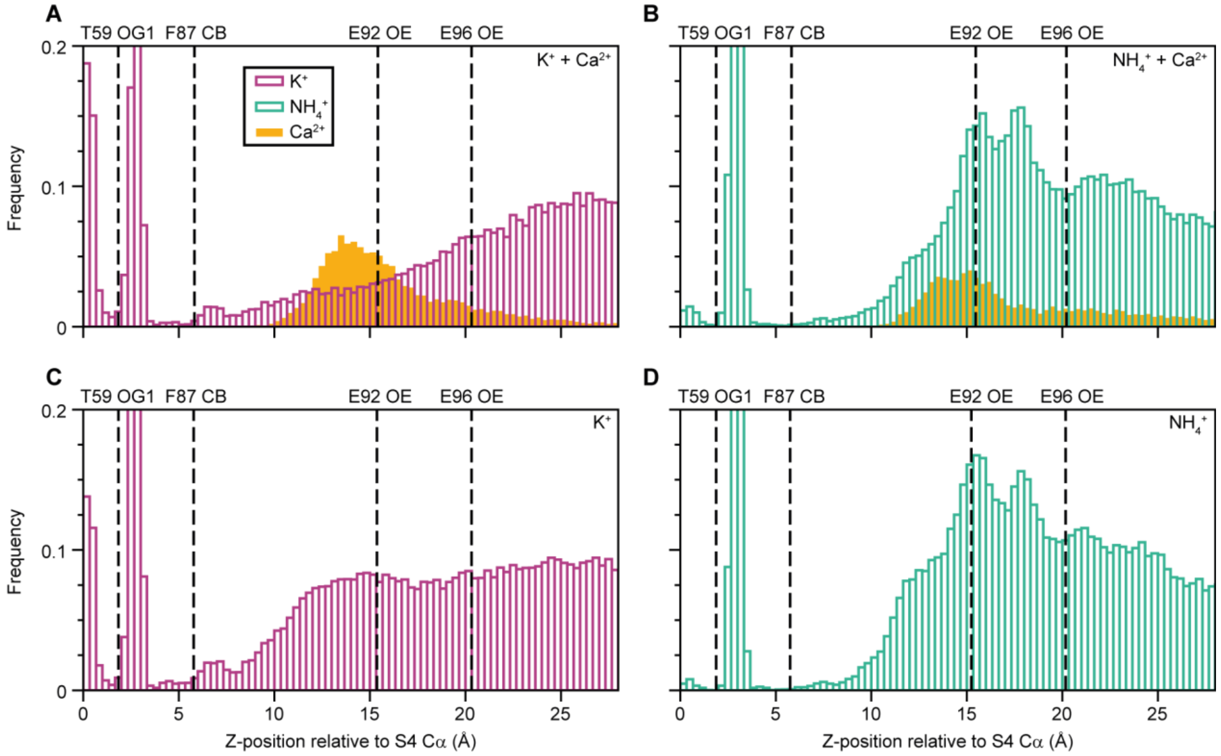

Figure S3. Ca^{2+} binding in the MthK cavity without voltage. Ion densities in the cavity relative to T59 from MD simulations without voltage over 10 replicates (A) KCl + $CaCl_2$, (B) NH_4Cl + $CaCl_2$,(C) KCl,(D) NH_4Cl.

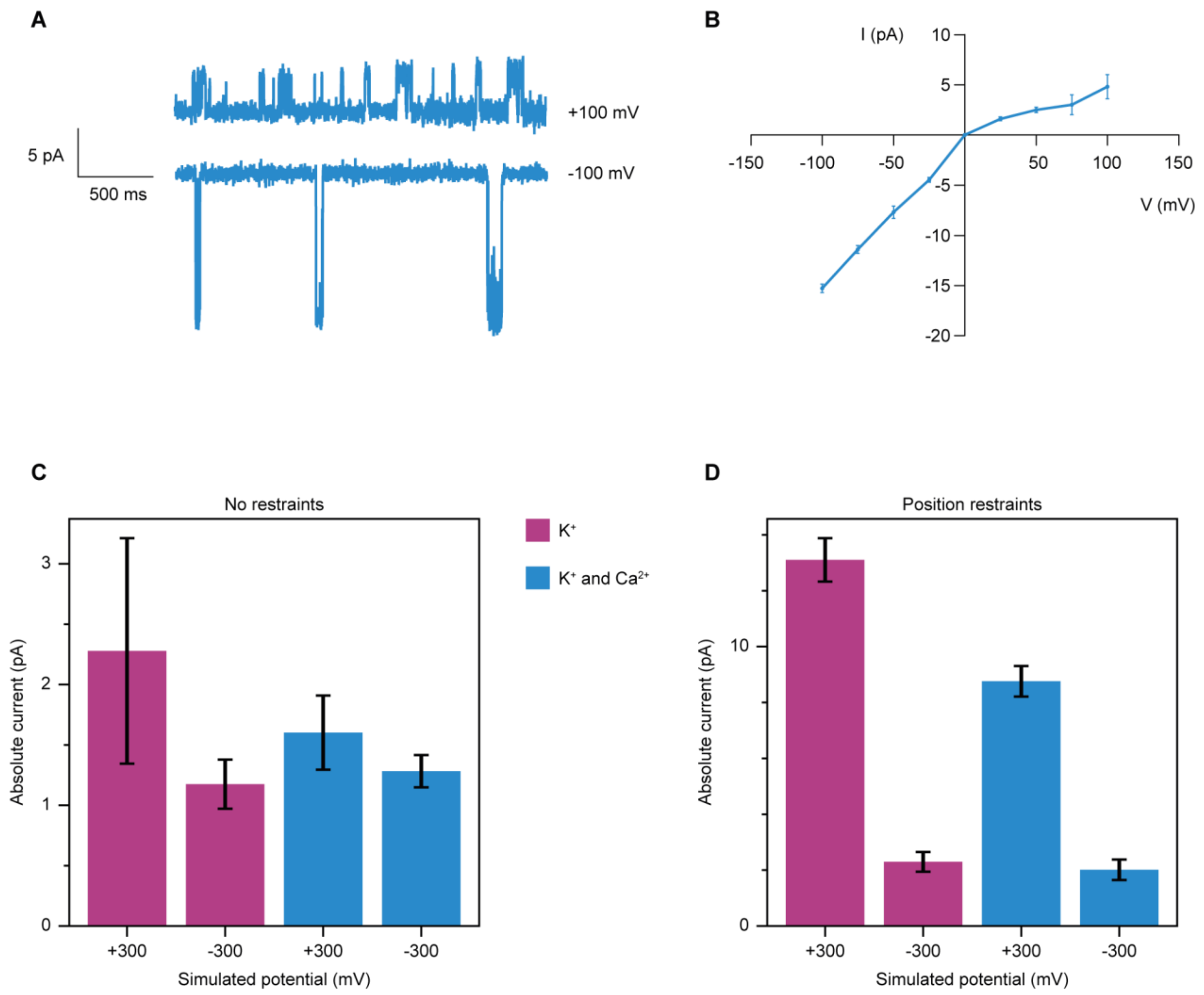

Figure S4. Experimental and simulated electrophysiology. (A) Examples of permeation events observed in electrophysiology experiments at +/- 100 mV. (B) I-V curve of MthK pore domain in the presence of 10 mM Ca^{2+}. Data points are mean and +/- standard error of the mean from multiple outbursts of one bilayer experiment. (C and D) Absolute simulated conductance with an applied voltage of +/- 300 mV with and without the addition of 30 mM Ca^{2+}. (C) Without restraints and (D) with position restraints applied to backbone atoms of residues 86-98. Error bars in (C) and (D) represent standard error of the mean over 10 independent replicates.

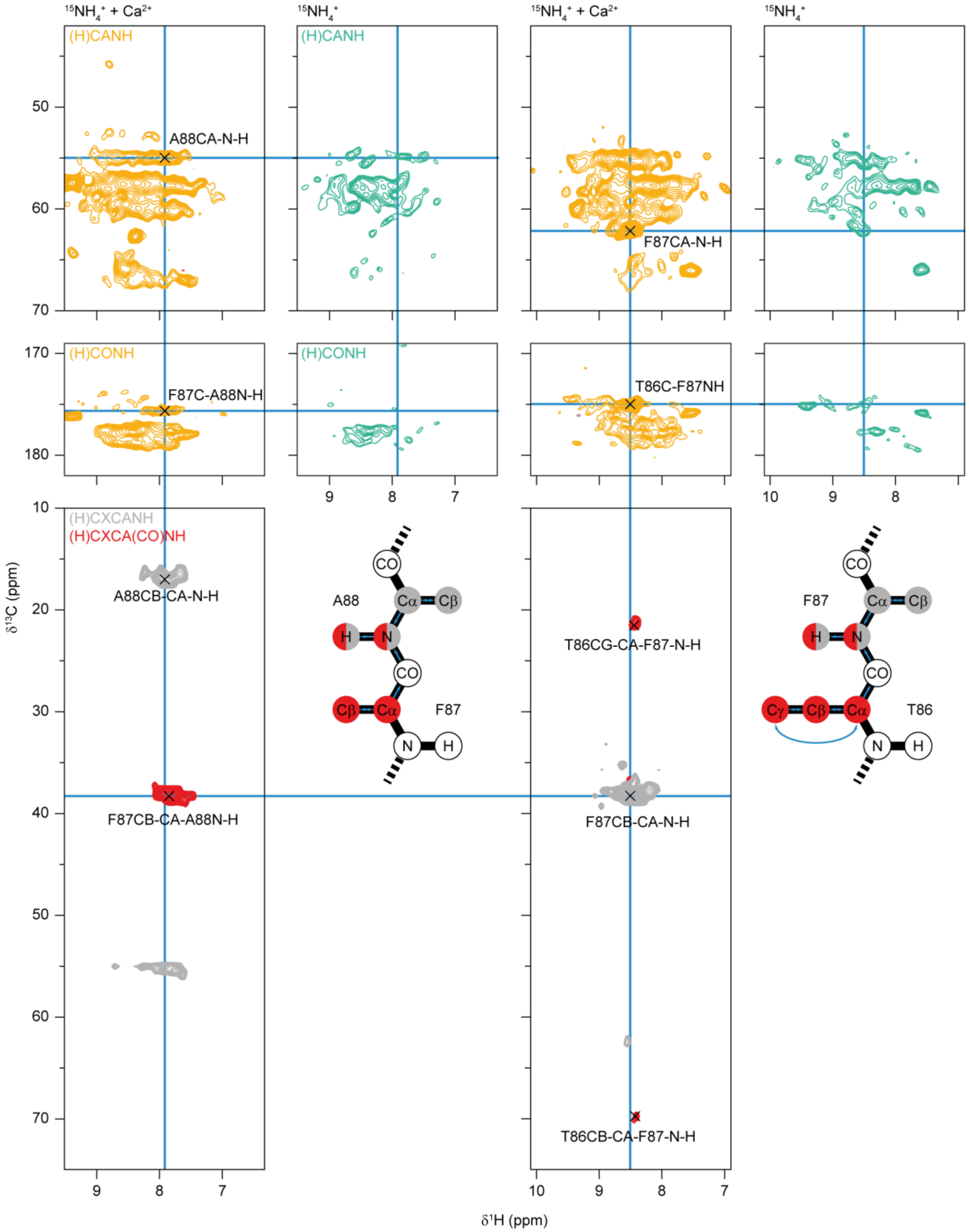

Figure S5. Example strip plots for the assignment of residues around the F87 Ca^{2+} binding site. Top, 2D CH strip plots of (H)CANH and (H)CONH (orange for the sample with 100 mM $^{15}NH_4^+$ and 10 mM Ca^{2+}, green for the sample with only $^{15}NH_4^+$) taken at the ^{15}N chemical shift of A88 (left) and F87 (right). Bottom, 2D CXH strip plots of 4D (H)CXCANH (grey) and (H)CXCA(CO)NH recorded on a sample with 100 mM $^{15}NH_4^+$ and 100 mM Ca^{2+}. The CXH strips of the (H)CXCANH are taken at the ^{15}N and ^{13}CA chemical shifts of residue i (A88 - left and F87 - right), while the CXH strip plots of the (H)CXCA(CO)NH are taken at the ^{15}N chemical shift of residue i (A88 - left, F87 - right) and the ^{13}CA chemical shift of residue i-1 (F87 - left, T86 - right). See also the schematic drawings indicating the atoms for which the peaks are observed in the 4D spectra.

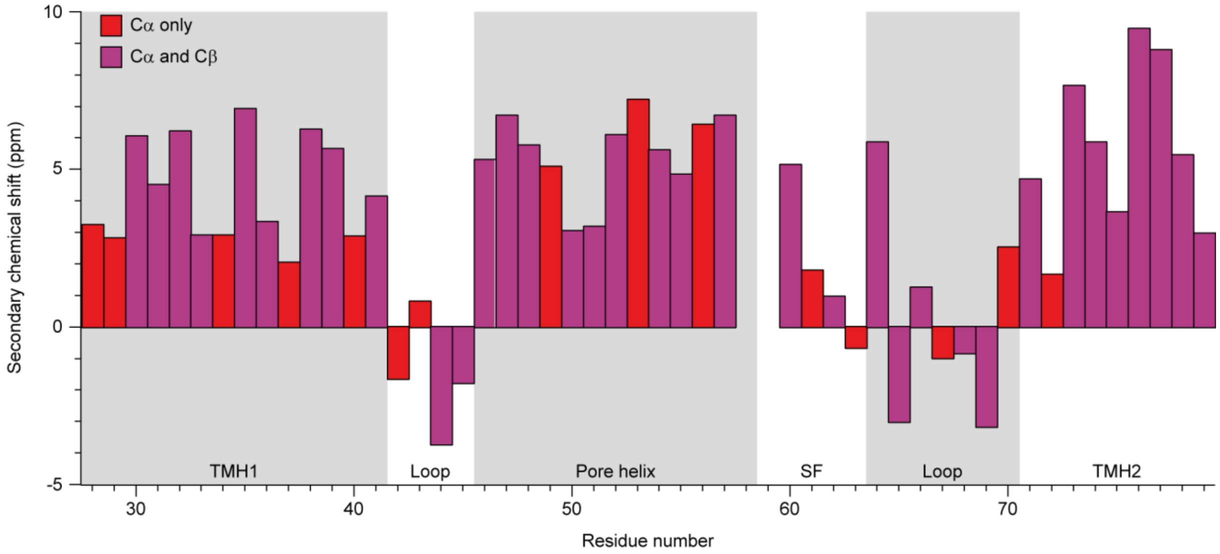

Figure S6. Secondary chemical shifts based on chemical shift assignments from ^{13}C detected solid-state NMR data (Table S1). The secondary chemical shifts were calculated as ($C\alpha_{observed}$ - $C\alpha_{random\ coil}$) - ($C\beta_{observed}$ - $C\beta_{random\ coil}$). Positive values are indicative of α-helical stuctures, negative values of β-strands and values around zero of coils. Only $C\alpha$ chemical shifts were used for calculations of glycines and residues for which no $C\beta$ assignments were available (shown as red bars).

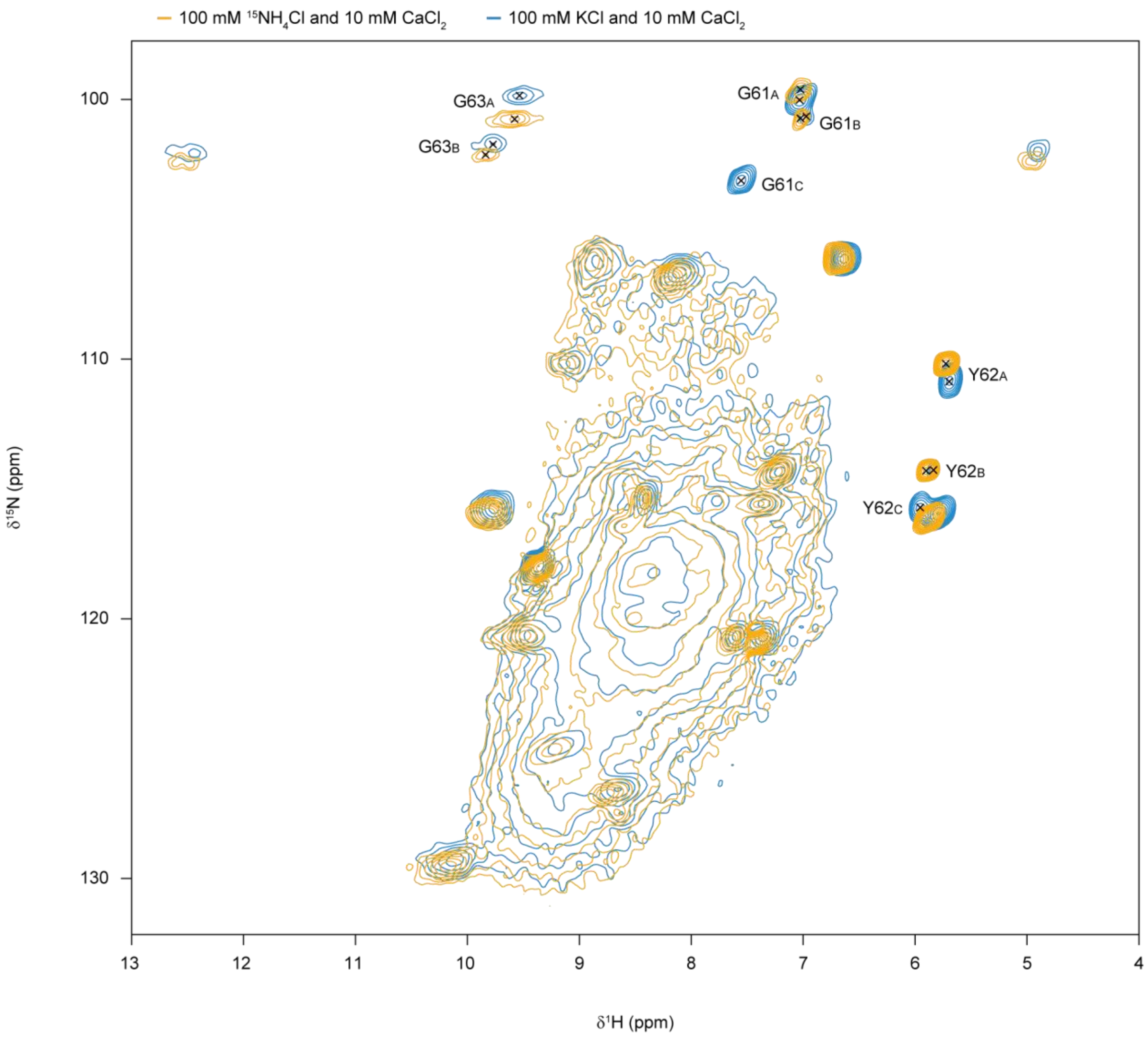

Figure S7. Comparison of samples with K^+ and $^{15}NH_4^+$. 2D (H)NH spectra of MthK with $^{15}NH_4^+$ (orange) and K^+ ions (blue). Both samples contain 10 mM Ca^{2+} ions. The different conformations of the selectivity filter residues are labelled (with "A","B","C").

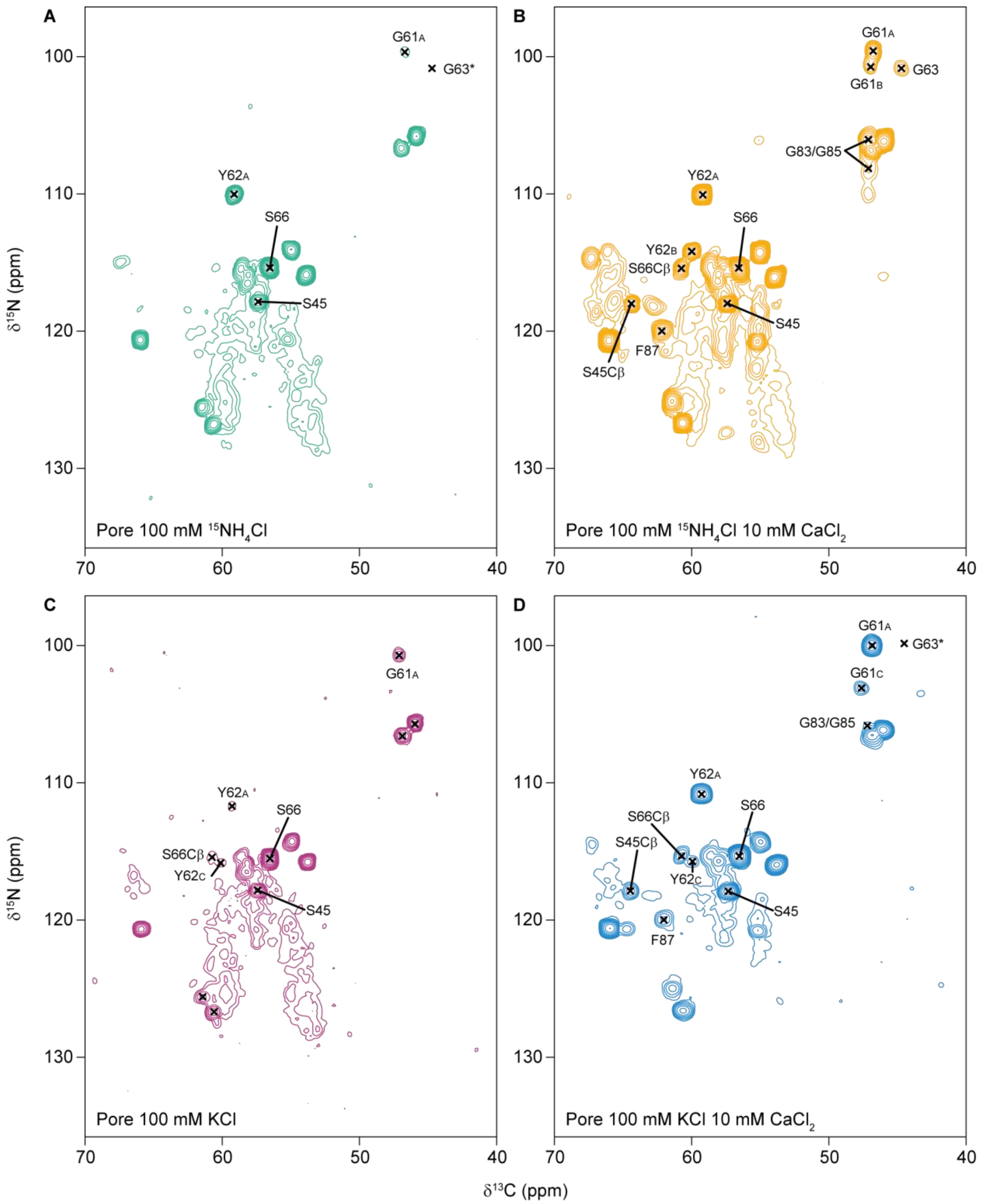

Figure S8. 2D NCA projections from ^{1}H detected 3D (H)CANH spectra of MthK pore domain with 100 mM $^{15}NH_4Cl$ (A, green), 100 mM $^{15}NH_4Cl$ + 10 mM $CaCl_2$ (B, orange), 100 mM KCl (C, purple), and 100 mM KCl + 10 mM $CaCl_2$ (D, blue). Peaks from the selectivity filter residues and other residues that are affected by Ca^{2+} are labelled. This includes serine N-CB peaks, that appear with high intensity in the presence of Ca^{2+} ions, and residues around the F87 Ca^{2+} ion binding site.

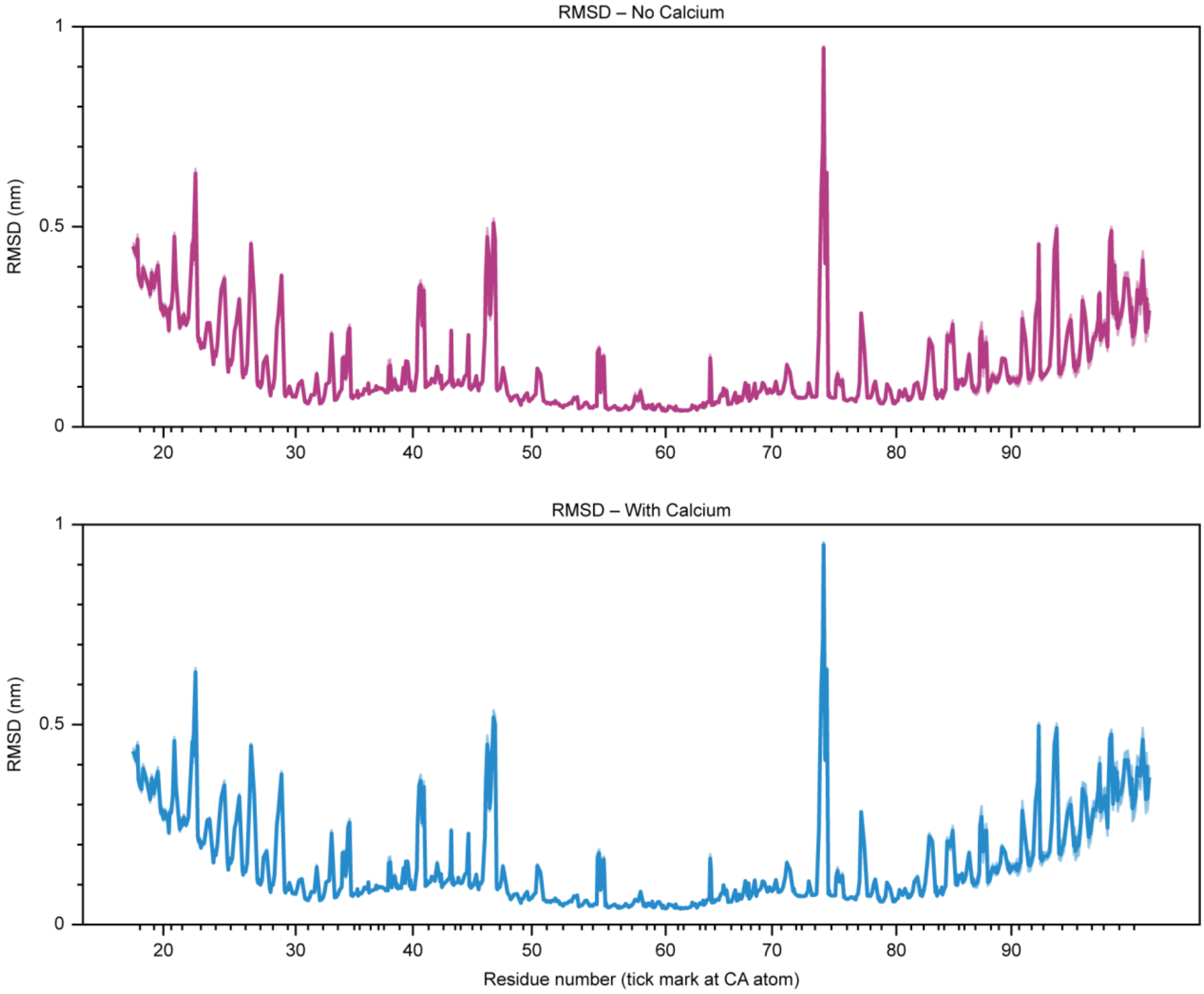

Figure S9. RMSD plots for MD simulations of MthK with K^+, without (top, purple) and with (bottom, blue) Ca^{2+}.

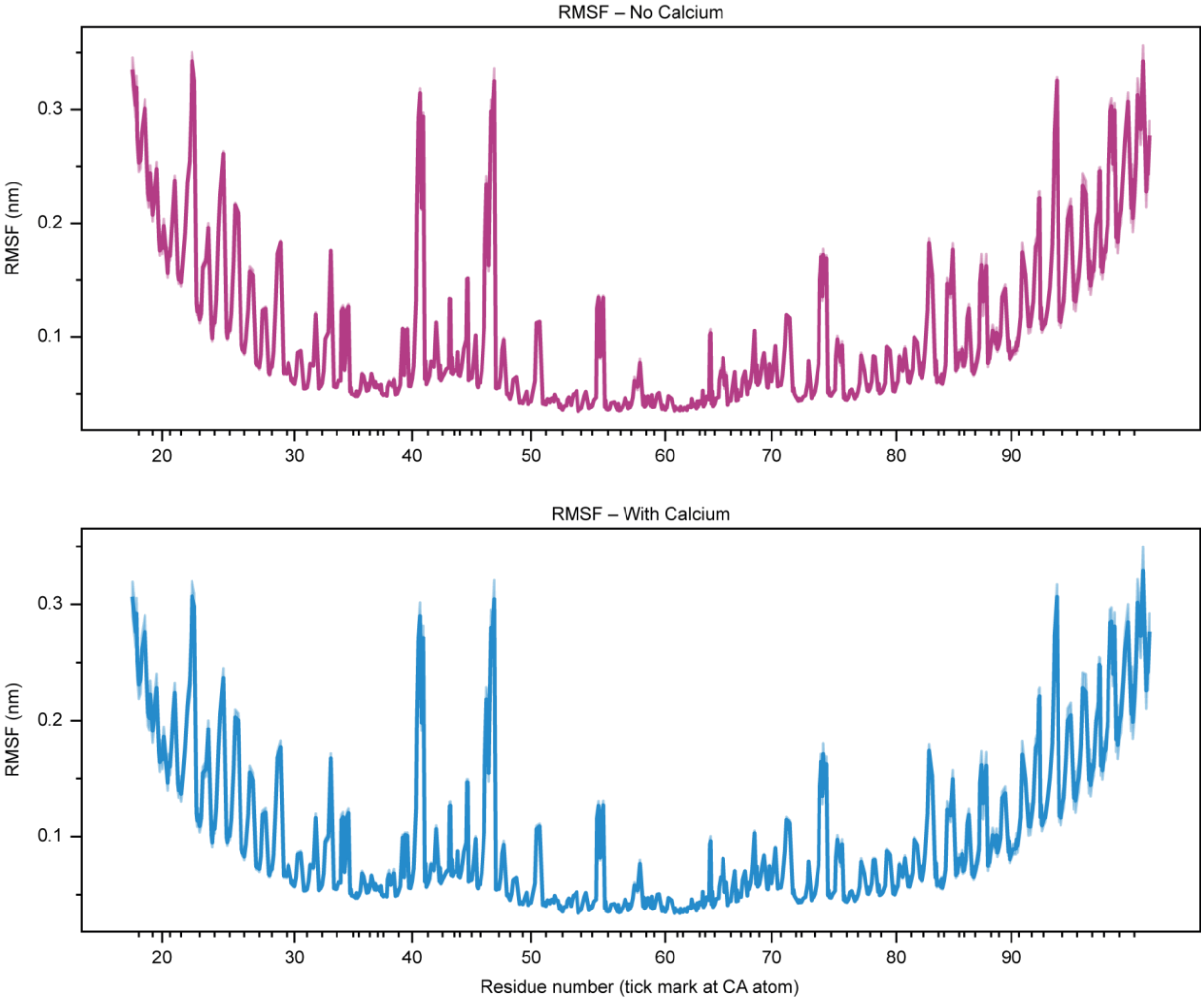

Figure S10. RMSF plots for MD simulations of MthK with K^+, without (top, purple) and with (bottom, blue) Ca^{2+}.

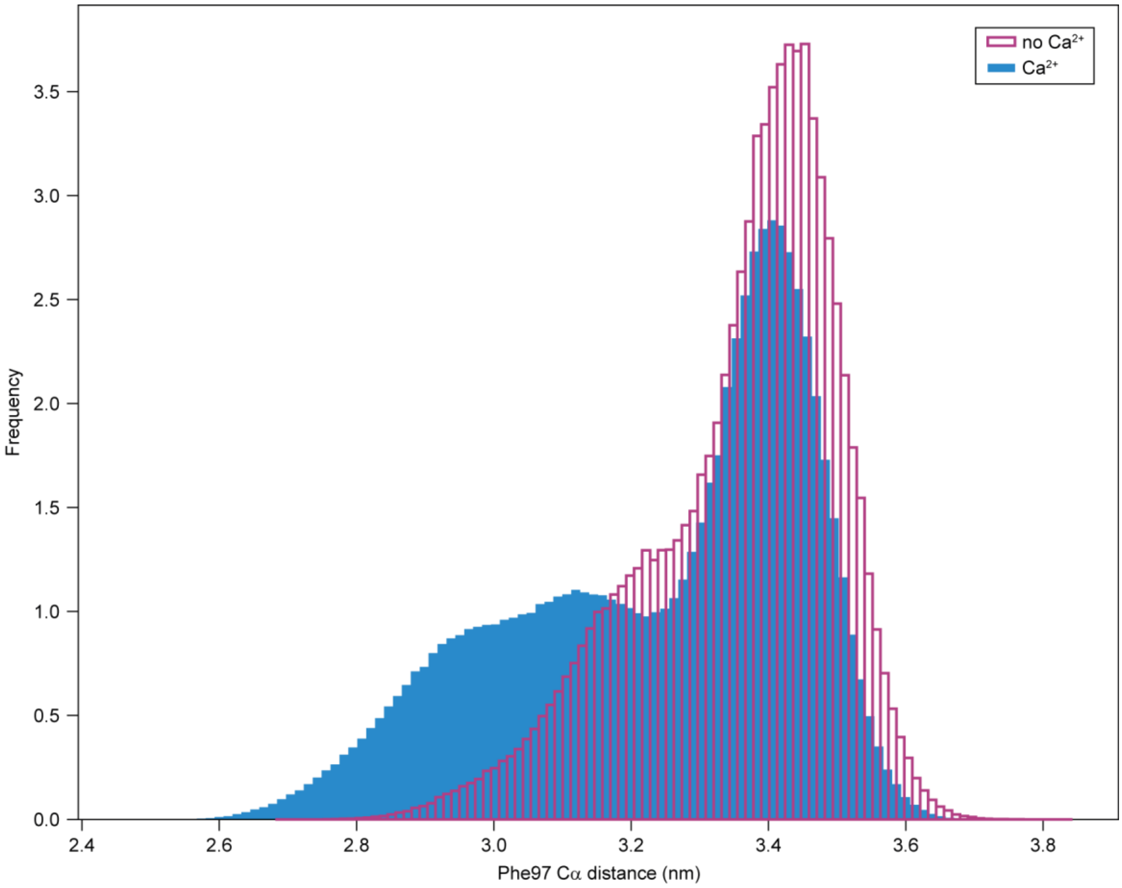

Figure S11. Lower gating opening distances. Mean distance between opposing F97 Cα atoms. Averaged over 50 5 μs simulations starting from 5 different initial opening distances.

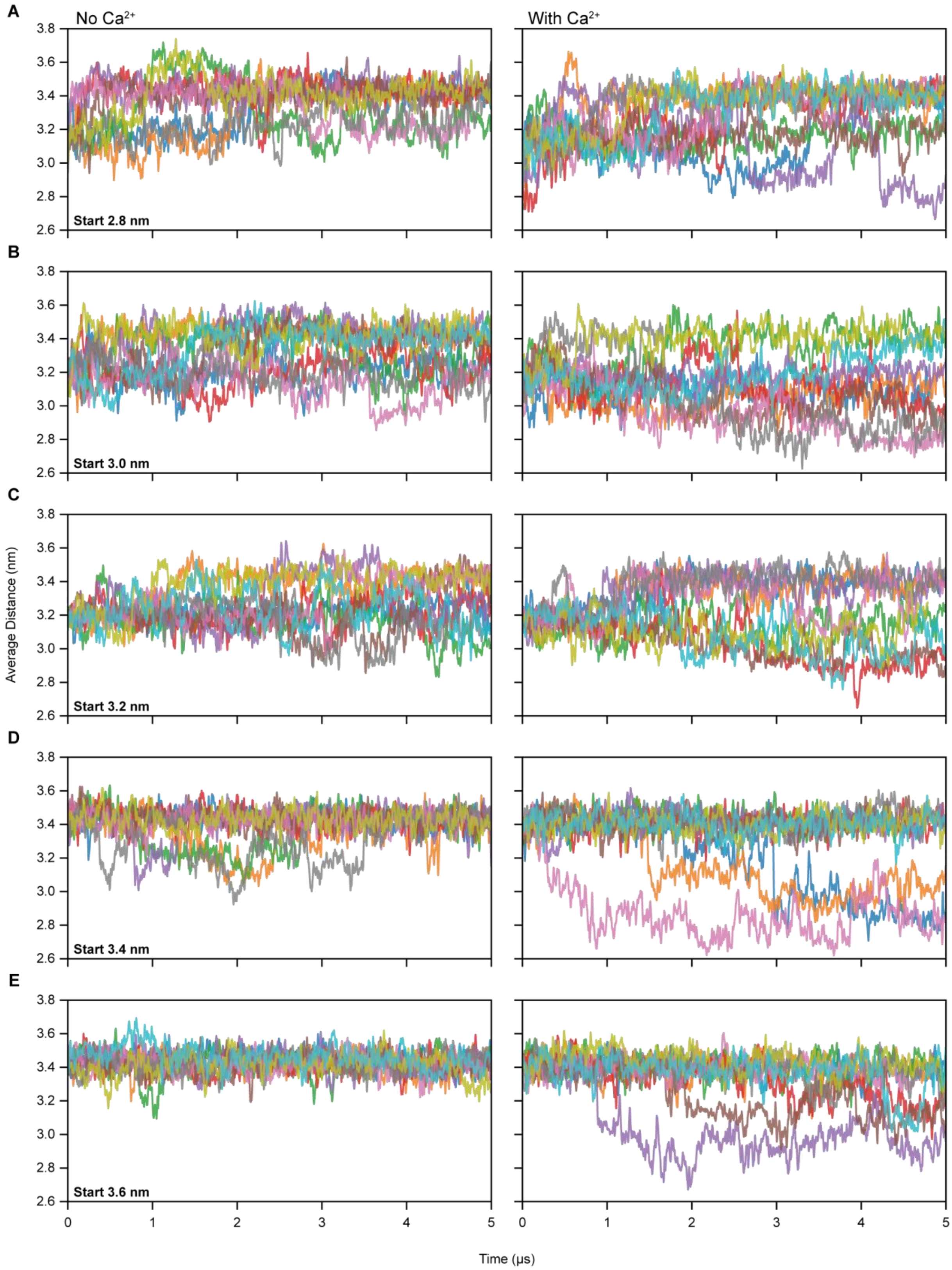

Figure S12. Smoothened timetraces of the average F97 Cα distance between opposing subunits. Each subplot shows traces from a specific starting condition based on the initial F97 Cα distance and whether calcium is included.

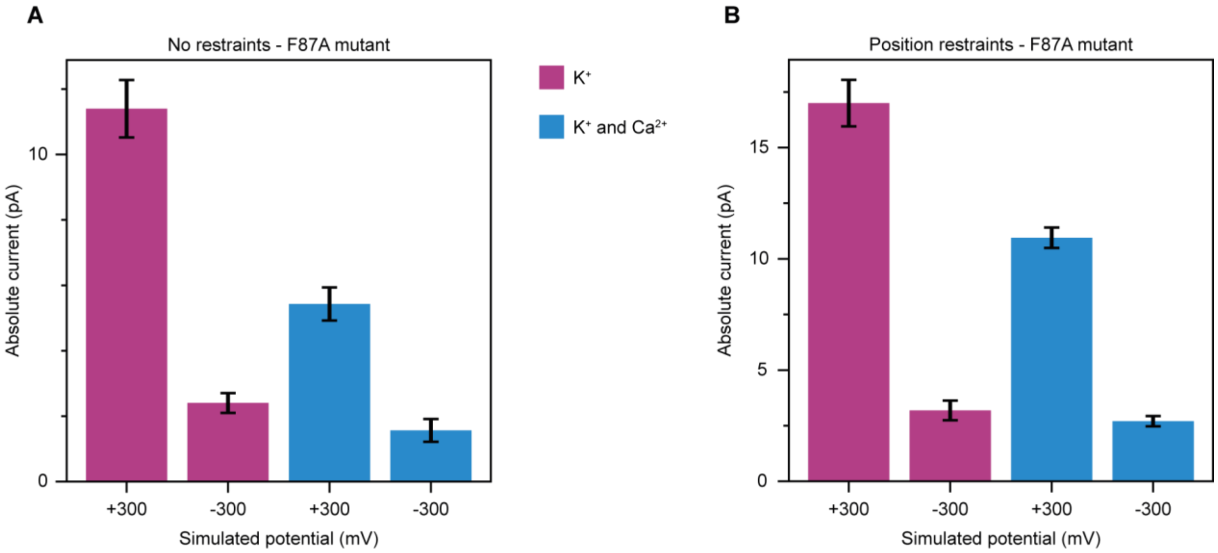

Figure S13. Simulated electrophysiology of MthK F87A. (A and B) Absolute simulated conductance with an applied voltage of +/- 300 mV with and without the addition of 30 mM Ca^{2+}. (A) Without restraints and (B) with position restraints applied to backbone atoms of residues 86-98. Error bars in (A) and (B) represent standard error of the mean over 10 independent replicates.

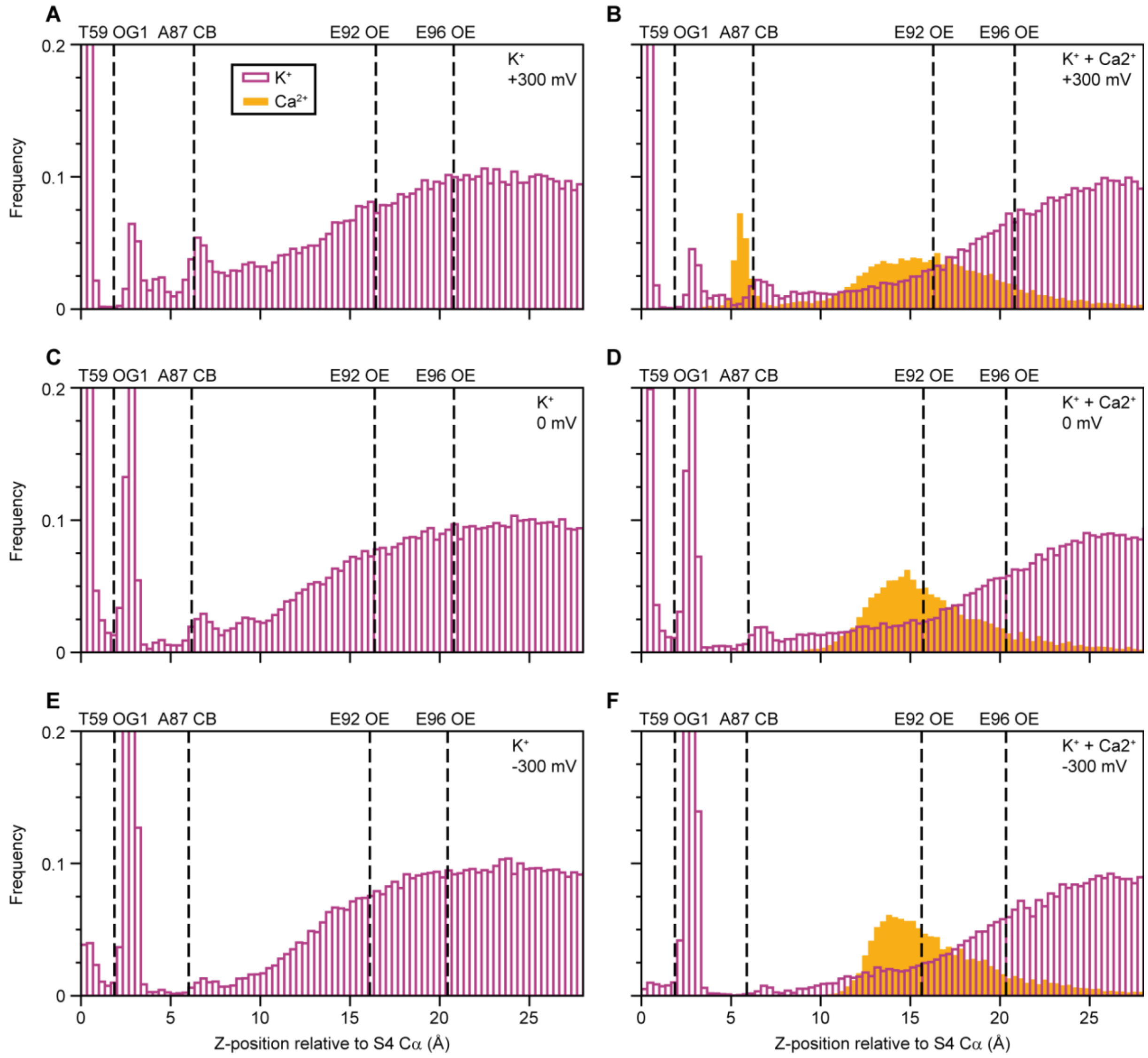

Figure S14. Ion densities in the MthK F87A cavity relative to T59 from MD simulations under different voltages with and without Ca^{2+} (A) KCl at 300 mV. (B) KCl + $CaCl_2$ at 300 mV. (C) KCl at 0 mV. (D) KCl + $CaCl_2$ at 0 mV. (E) KCl at -300 mV. (F) KCl + $CaCl_2$ at -300 mV.

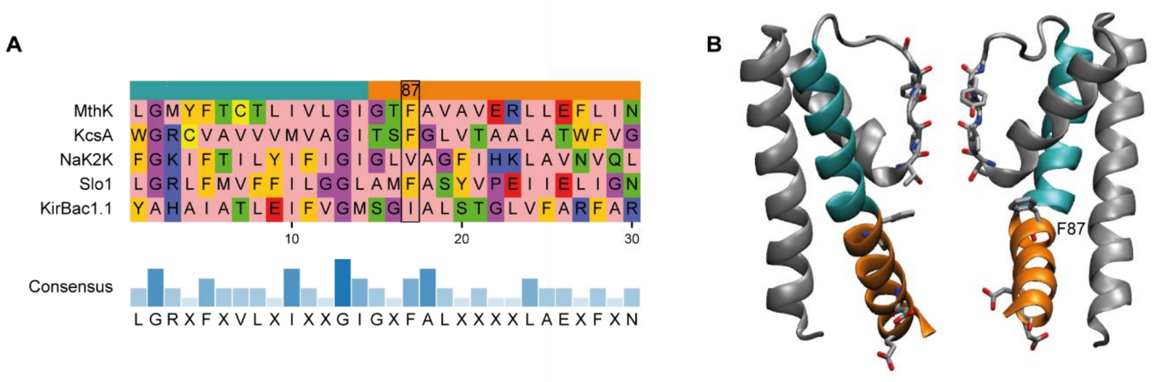

Figure S15. Multiple sequence alignment of the pore lining helix (TMH2 in MthK). (A) Sequence alignment comparing MthK, KcsA, NaK2K, human Slo1, and KirBac1.1. The residues compared correspond to residues 71 to 100 in MthK. (B) Open MthK structure highlighting the top of the helix in cyan and the residues lining the cavity in orange. F87 in MthK is indicated in both (A) and (B).

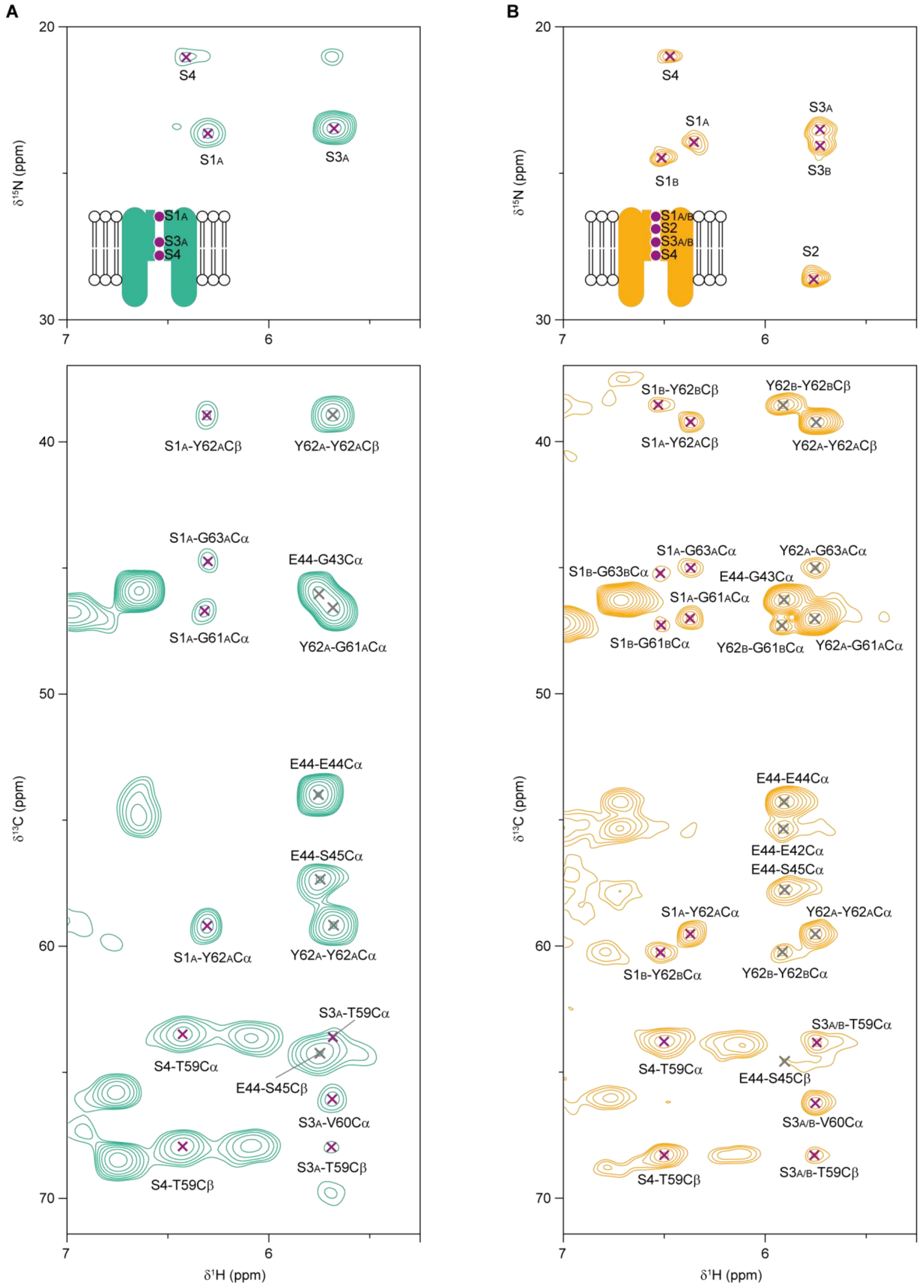

Figure S16. Detection of bound $^{15}NH_4^+$ ions in the MthK pore domain. ^{1}H detected INEPT-based 2D (H)NH spectra of $^{15}NH_4^+$ (top) and CP-based (H)CXH spectra (bottom) recorded on $^2H^{13}C^{15}N$ labelled MthK pore domain samples with 100 mM $^{15}NH_4Cl$ (A, green spectra) and 100 mM $^{15}NH_4Cl$ + 10 mM $CaCl_2$ (B, orange spectra). Peaks involving $^{15}NH_4^+$ ions are labelled with purple crosses and peaks between backbone atoms are labelled with dark grey crosses.

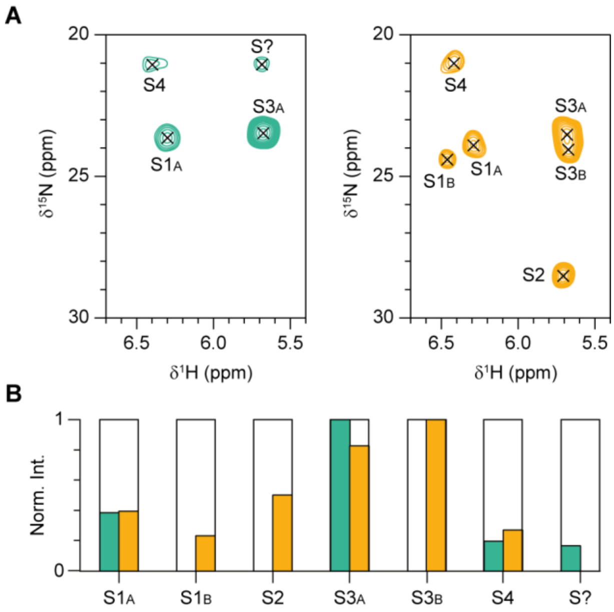

Figure S17. Comparison of peak intensities for bound $^{15}NH_4^+$ ions. (A) ^{1}H detected INEPT-based 2D (H)NH spectra of $^{15}NH_4^+$ without (left, green spectrum) and with (right, orange spectrum) 10 mM $CaCl_2$ in the sample. (B) Peak intensities for all detected $^{15}NH_4^+$ peaks. The peak intensities are normalized to the peak with the highest intensity internally in each spectrum. Both spectra were recorded on a 600 MHz spectrometer, at 55 kHz MAS.

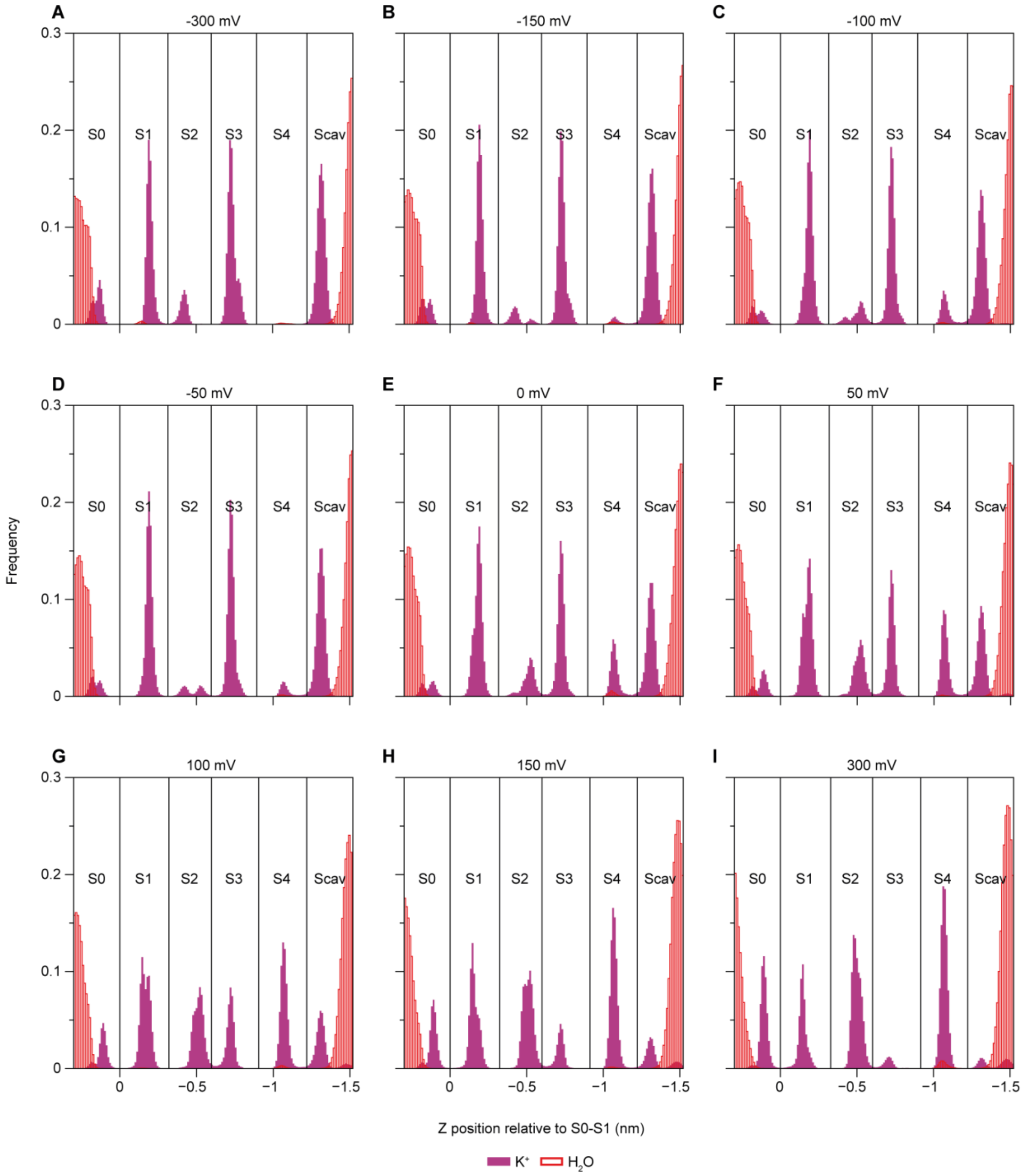

Figure S18. Influence of voltage on SF occupancy for simulations with K^+ ions. (A to I) K^+ and water occupancies at -300 to +300 mV, in steps of 50 mV. K^+ ions are labelled in purple and water in red.

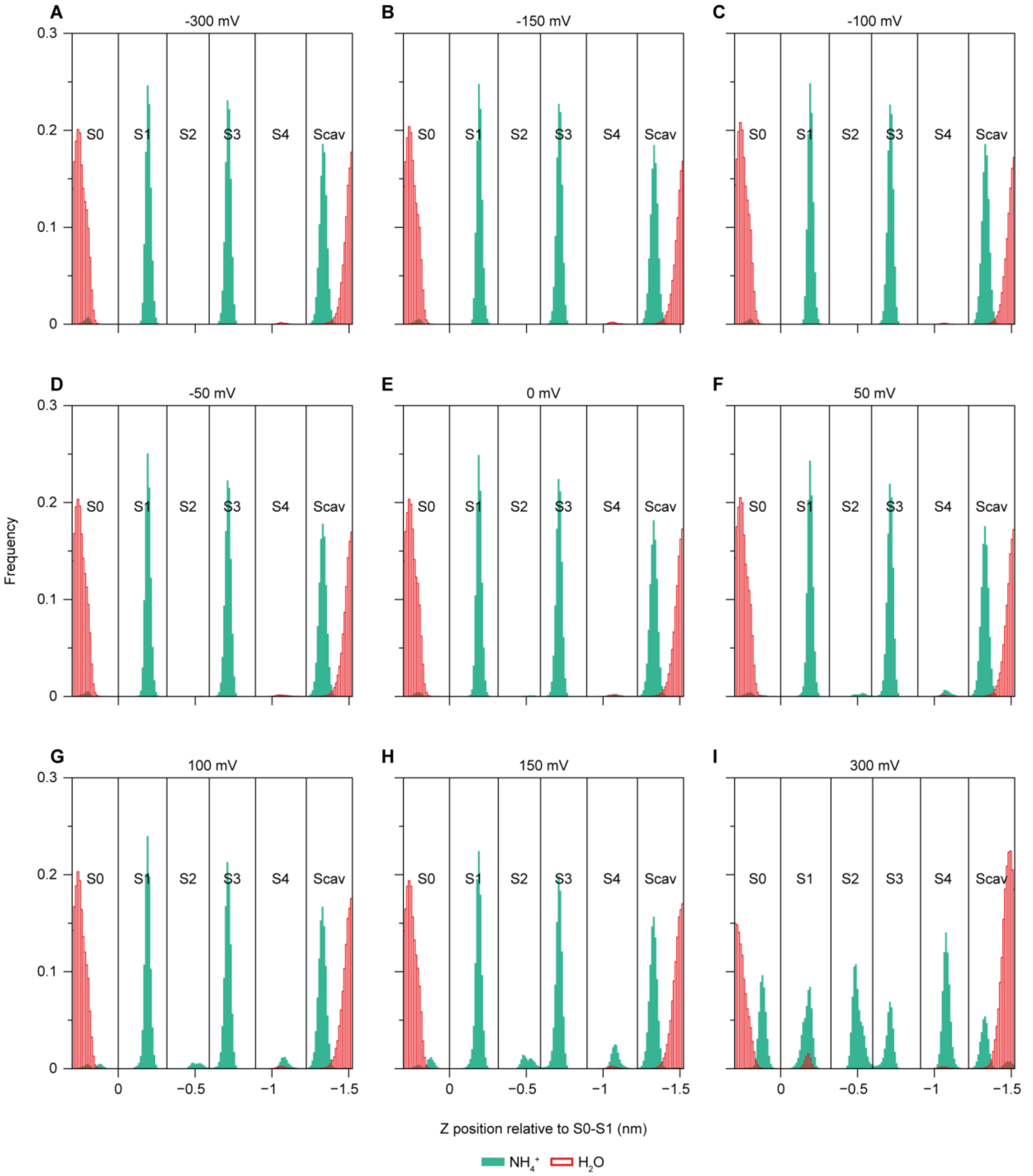

Figure S19. Influence of voltage on SF occupancy for simulations with NH_4^+ ions. (A to I) NH_4^+ and water occupancies at -300 to +300 mV, in steps of 50 mV. NH_4^+ ions are labelled in green and water in red.

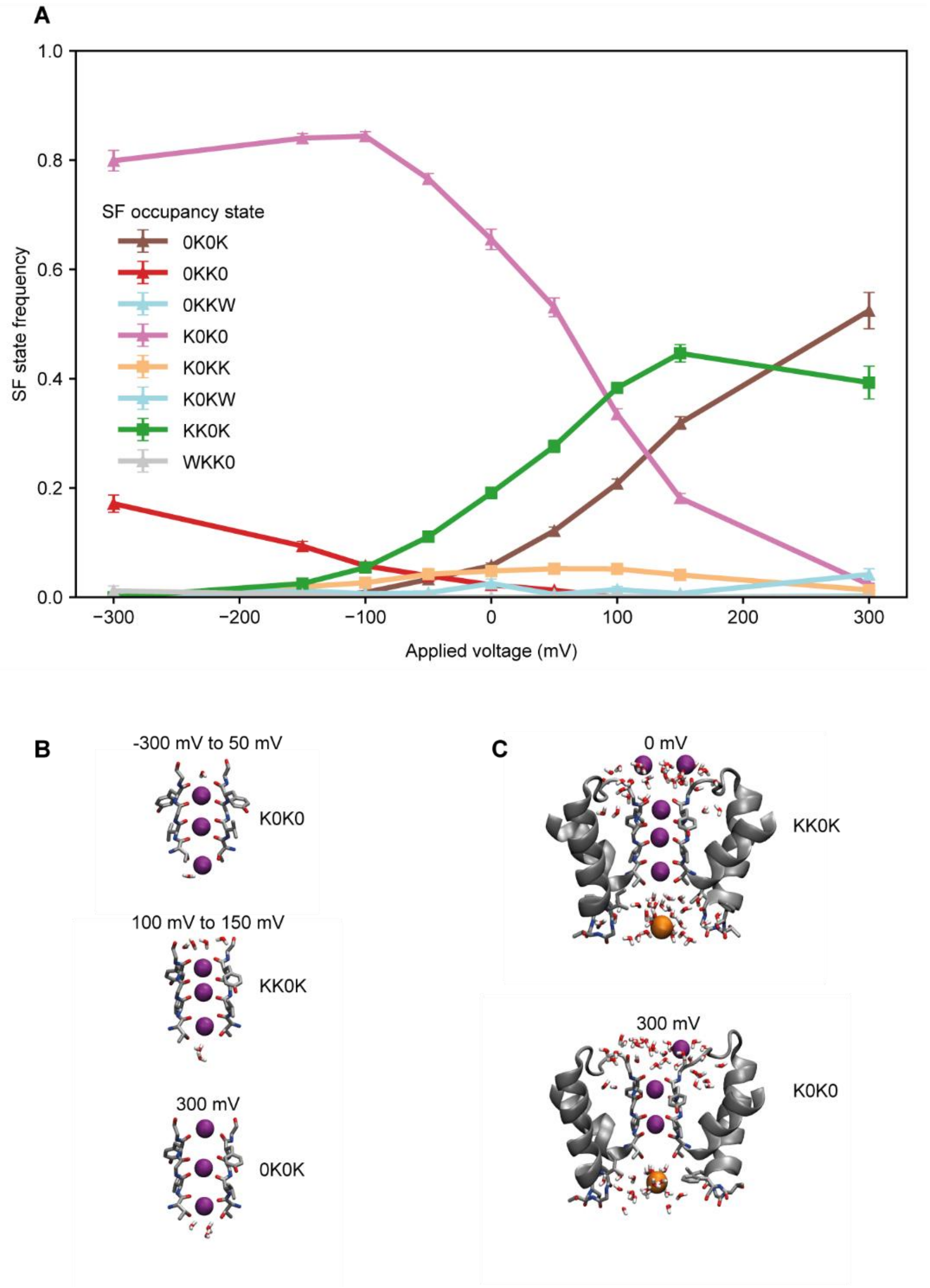

Figure S20. Influence of voltage and Ca^{2+} ions on SF occupancy states for simulations with K^+. (A) SF occupancy in sites S1-S4 under different applied voltages, without Ca^{2+}. K, W, and 0 represent presence of K^+, water, or a vacancy respectively. Error bars represent standard error of the mean over 10 independent replicas. (B) Representative snapshots of the most common SF occupancy states for different voltages in simulations without Ca^{2+}. (C) Representative snapshots of SF occupancy states when Ca^{2+} is bound below the SF for 0 and 300 mV.

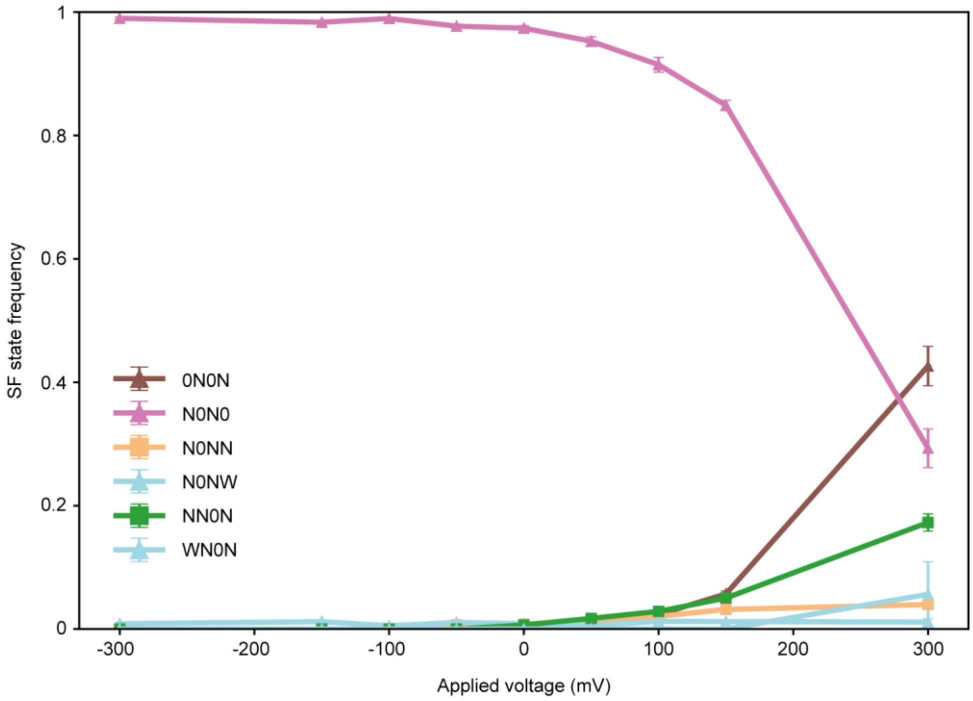

Figure S21. Influence of voltage on SF occupancy for simulations with NH_4^+. SF occupancy in sites S1-S4 under different applied voltages, without Ca^{2+}. N, W, and 0 represent presence of NH_4^+, water, or a vacancy respectively. Error bars represent standard error of the mean over 10 independent replicas.

Table S1. Chemical shift assignments (deposited in the BMRB under ID: 53315) based on ^{13}C detected experiments on $^{13}C^{15}N$ labelled MthK pore domain with 100 mM KCl. Values are in ppm and are referenced externally using DSS.

Residue number	Residue type	N	C	CA	CB
28	LEU	-	177.99	58.15	-
29	ALA	120.39	178.92	55.62	-
30	VAL	116.65	177.45	66.87	31.40
31	ILE	119.46	179.75	64.37	37.54
32	ILE	123.55	176.32	66.30	37.77
33	TYR	120.54	177.99	61.51	39.59
34	GLY	103.64	174.94	48.42	
35	THR	116.98	176.75	67.41	68.98
36	ALA	121.02	179.38	55.12	18.08
37	GLY	104.62	174.29	47.56	
38	PHE	123.92	177.84	63.39	38.61
39	HIS	115.72	178.09	58.10	26.54
40	PHE	115.12	176.85	60.89	
41	ILE	117.11	176.83	64.53	38.08
42	GLU	114.62	177.40	55.21	
43	GLY	106.16	174.69	46.32	
44	GLU	115.34	175.92	53.53	30.60
45	SER	118.66	175.45	57.52	64.93
46	TRP	126.38	177.89	61.84	28.43
47	THR	115.81	175.86	67.70	69.48
48	VAL	120.97	177.67	66.27	31.10
49	SER	115.97	176.49	63.51	
50	LEU	128.24	178.74	57.70	42.15
51	TYR	122.09	176.16	60.67	38.48
52	TRP	121.36	178.94	61.91	27.70
53	THR	121.36	176.66	68.83	
54	PHE	119.16	177.16	63.88	39.75
55	VAL	117.80	178.11	66.36	32.10
56	THR	119.94	175.99	68.03	
57	ILE	114.90	175.23	64.97	35.95
60	VAL	122.05	177.28	66.15	31.58
61	GLY	101.16	173.28	47.30	
62	TYR	112.08	178.08	59.82	39.84
63	GLY	100.15	175.17	44.80	
64	ASP	120.69	175.48	55.42	36.25
65	TYR	115.41	174.34	57.32	41.38
66	SER	115.67	169.69	56.66	61.00
67	PRO		176.21	62.48	
68	SER	116.17	174.52	58.41	64.88
69	THR	115.30	172.79	58.56	70.26
70	PRO		178.27	66.04	
71	LEU	117.24	178.77	58.39	41.20
72	GLY	106.60	177.98	47.17	
73	MET	127.40	177.62	61.05	31.09
74	TYR	118.41	178.91	63.46	38.59
75	PHE	119.84	178.87	60.21	38.05
76	THR	122.12	175.67	68.71	67.74
77	VAL	121.71	177.26	68.45	30.25
78	THR	108.43	174.78	66.05	69.09
79	LEU	123.37	178.66	57.43	41.95

Table S2. Chemical shift assignments (deposited in the BMRB under ID: 53314) based on ^{1}H detected experiments on 100% H_2O back-exchanged $^{2}H^{13}C^{15}N$ labelled MthK pore domain with 100 mM $^{15}NH_4Cl$ and 10 or 100 mM $CaCl_2$. Values are in ppm and are referenced externally using DSS.

Residue number	Residue type	H	N	C	CA	CB	CG
38	PHE			177.88	63.05	38.24	
39	HIS	7.81	115.69	177.93	57.87	26.73	
41	ILE			176.83	64.17		
42	GLU	7.24	114.35	177.66	54.99	27.56	35.73
43	GLY	6.67	106.59	174.79	46.04		
44	GLU	5.77	116.06	176.02	53.72	29.75	34.18
45	SER	9.37	117.92	175.67	57.38	64.67	
46	TRP	9.16	124.88	177.77	61.51	28.14	
47	THR	8.60	114.83	175.72	67.42	69.29	20.47
48	VAL	7.62	120.67	177.54	66.05	30.13	22.48
49	SER			176.27	63.23		
50	LEU	8.63	127.68		57.53	41.35	
59	THR			171.75	63.55	67.96	
60A	VAL			176.63	66.09		
61A	GLY	6.96	99.63	173.28	46.75		
62A	TYR	5.66	110.01	178.63	59.18	38.87	
63	GLY	9.59	100.86	175.22	44.64		
64	ASP	9.43	120.99	175.58	55.11	35.77	
65	TYR	7.32	115.76	174.19	57.37	40.27	
66	SER	8.37	115.18	169.82	56.60	60.84	
67	PRO			175.82	62.33	31.50	
68	SER	9.75	115.73	174.65	58.00	64.71	
69	THR	8.58	115.54	173.21	58.57		20.99
70	PRO			178.24	65.84		
71	LEU	8.70	116.51	178.57	58.16	40.22	
72	GLY	8.13	106.61	177.87	46.86		
73	MET	8.69	126.67	177.66	60.72	30.13	32.34
74	TYR	8.34	118.11	178.75	63.08	37.57	
75	PHE	9.28	119.74		60.03	38.14	
86	THR			175.16	66.15	69.76	21.00
87	PHE	8.48	120.21	175.55	62.11	38.22	
88	ALA	7.81	118.71	180.66	55.05	16.94	
89	VAL	7.77	116.68	177.16	64.71	33.66	
60B	VAL			175.63			
61B	GLY	6.97	101.06	174.38	46.83		
62B	TYR	5.83	114.27	178.53	60.01	38.21	

Table S3. Chemical shift assignments of the different conformations of the selectivity filter residues for MthK pore domain with K^+ ions. Assignments are based on ^1H detected (H)NH, (H)CANH, and (H)CONH spectra recorded on 100% H_2O back-exchanged $^2H^{13}C^{15}N$ labelled MthK pore domain with 100 mM KCl and 10 mM $CaCl_2$. Values are in ppm and are referenced externally using DSS.

Residue number	Residue type	H	N	C	CA
60A	VAL			177.02	
61A	GLY	7.01	100.01	173.26	46.88
62A	TYR	5.68	110.83	178.21	59.28
63A	GLY	9.55	99.78		44.64
60B	VAL			175.58	
61B	GLY	6.96	100.66	174.47	
62B	TYR	5.83	114.22		
63B	GLY	9.77	101.72		
60C	VAL			179.29	
61C	GLY	7.55	103.10	174.60	47.63
62C	TYR	5.94	115.68		59.94

Table S4. Chemical shift assignments of $^{15}NH_4^+$ ions bound in the selectivity filter of MthK pore domain. Assignments are based on a 2D (H)NH INEPT spectrum and 2D (H)COH and (H)CXH CP spectra recorded on a sample with 100 mM $^{15}NH_4Cl$ and 10 mM $CaCl_2$. Values are in ppm and are referenced externally using DSS.

Ion binding site	H	N
S1A	6.36	23.94
S1B	6.51	24.48
S2	5.76	28.61
S3A	5.74	23.53
S3A	5.73	24.06
S4	6.47	21.03

Supplementary Movie 1. Typical Ca^{2+} block of the MthK pore domain observed in a 250 ns segment of MD simulations in the presence of KCl and $CaCl_2$ under positive voltage. The top left plot shows the distance between the bottom (side chain of T59) of the SF and the closest Ca^{2+} ion below the SF. The bottom left plot counts cumulative permeation; jumps indicate permeation events. The movie on the right shows Ca^{2+} ions (in orange) and K^+ ions (in purple) in and near the MthK pore. Side chains for residues near ion binding sites are shown in more detail; residues 59 to 63 for the SF, residues 84 to 87 for the Scav binding site and residues 92, 93 and 96 for the lower binding site. Only 2 out of 4 monomers are shown for clarity.
